# HSP70 is a chaperone for IL-33 activity in chronic airway disease

**DOI:** 10.1172/jci.insight.193640

**Published:** 2025-06-24

**Authors:** Omar A. Osorio, Heather E. Raphael, Colin E. Kluender, Ghandi F. Hassan, Lucy S. Cohen, Deborah F. Steinberg, Ella Katz-Kiriakos, Morgan D. Payne, Ethan M. Luo, Jamie L. Hicks, Derek E. Byers, Jennifer Alexander-Brett

**Affiliations:** 1 Department of Medicine, Division of Pulmonary and Critical Care Medicine, and; 2 Department of Pathology and Immunology, Washington University School of Medicine, St. Louis, Missouri, USA.

**Keywords:** Immunology, Pulmonology, COPD, Chaperones, Cytokines

## Abstract

IL-33 is a key driver of type 2 inflammation and implicated in pathology of chronic obstructive pulmonary disease (COPD) and asthma. However, the mechanism for IL-33 secretion and regulation in the context of chronic airway disease is poorly understood. We previously reported an airway disease–associated isoform IL-33^Δ34^ that escapes nuclear sequestration and is tonically secreted from epithelial cells. Here, we describe how this IL-33^Δ34^ isoform interacts with HSP70 within cells and is targeted to secretory organelles through coordinated binding to phosphatidylserine (PS) and delivered to compartments for unconventional protein secretion (CUPS). Once secreted, extracellular HSP70 (eHSP70) in complex with IL-33^Δ34^ stabilizes the cytokine by inhibiting oxidation and degradation, which results in enhanced IL-33^Δ34^-receptor binding and activity. We further find evidence that IL-33 along with mediators of the proteostasis network HSP70, HSP90, and the Chaperonin Containing TCP1 (CCT) complex are dysregulated in human chronic airway disease. This phenomenon is reflected in the differential extracellular vesicle (EV) proteome in bronchial wash from COPD and asthma samples, which could mark disease activity and potentiate IL-33 function. This study confirms proteostasis intermediates, chiefly HSP70, as chaperones for noncanonical IL-33 secretion and activity that may be amenable for therapeutic targeting in the chronic airway diseases COPD and asthma.

## Abstract 2

This study reveals a mechanism by which heat shock chaperones enhance inflammatory cytokine activity in airway disease pathogenesis. Analysis of lung specimens from patients with chronic obstructive pulmonary diseaes and asthma reveal a global dysregulation of protein quality control pathways that contribute to airway diseases.

## Graphical abstract

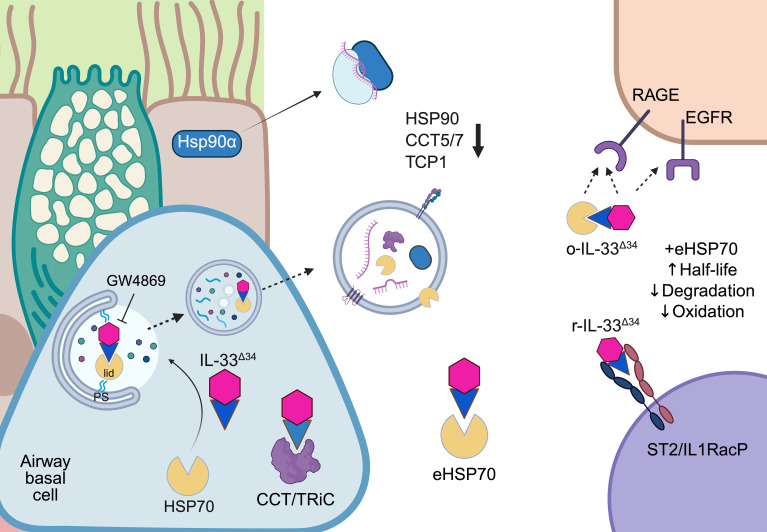

## Introduction

IL-33 is an epithelial-derived cytokine (1) that can polarize inflammatory responses toward a type 2 immune phenotype (2, 3). Support for IL-33 as a central mediator of human airway disease is derived from genome-wide association studies (4–6), and analysis of specimens from human asthma (7, 8) and patients with chronic obstructive pulmonary disease (COPD) (9–12). Several animal models of airway disease have proven the essential role of IL-33 expression and release as a trigger for airway disease in response to virus infection (9) and allergen challenge (12–14).

The mechanism of IL-33 secretion has been investigated under a variety of conditions, including in response to danger signals and under conditions of cell necrosis or infection models (15). We previously analyzed the mechanism of respiratory epithelial IL-33 secretion in COPD, focusing on airway basal cells as the primary cellular source of this cytokine in human lungs. We observed continuous secretion of disease-associated spliced IL-33 isoform from basal cells, and this secretion was dependent on the neutral sphingomyelinase-2–dependent (nSMase2-dependent) multivesicular endosome (MVE) pathway. We also found that IL-33 could be cosecreted while noncovalently bound to small extracellular vesicles (sEVs) (~100–150 nm diameter) that exhibited features consistent with exosomes of endosomal origin (12, 16). We found nSMase2 expression and activity to be increased in COPD-derived specimens and demonstrated attenuated IL-33–driven type 2 inflammation under conditions of nSMase2 blockade in an Alternaria model.

HSP70 (encoded by *HSPA1A*) (17) is an inducible member of the heat shock protein (HSP) family that broadly function as intracellular chaperones in: folding of nascent or misfolded proteins, translocating of polypeptides across organelle membranes, and targeting of irreparably damaged proteins for degradation by the ubiquitin-proteasome system or via autophagy (18–20). HSP70 is considered a sentinel guardian of the cellular proteome, given its expression is rapidly upregulated in response to cellular stress to help cells overcome proteotoxic stress and mitigate damage (21–23). The extracellular form of HSP70 (eHSP70) has been characterized as a DAMP that is thought to be released passively from dying or damaged cells or through various unconventional protein secretion mechanisms. eHSP70 can be released in soluble form through membrane channels, translocate across the plasma membrane, or be released from secretory-like organelles (24), endolysosomes (25), or as EV cargo (24). EVs are membrane delineated microparticles secreted by all cells either through outward budding directly from the plasma membrane (ectosomes) or through intraluminal vesicles (ILVs) formed within MVEs (26). These vesicles are collectively labeled EVs and function as platforms for intercellular communication in homeostasis and disease (26). In a pathogenic context, EVs from asthmatic bronchoalveolar lavage fluid (BALF) contain Leukotriene-generating enzymes (27), EVs from bronchial epithelial cells exposed to cigarette smoke extract (CSE) promote lung fibroblast differentiation into myofibroblasts (28), and IL-13–induced epithelial cells secrete EVs that can drive proliferation of monocytes (29, 30).

As a secreted protein, eHSP70 has been found dysregulated in multiple chronic inflammatory conditions (31). Likewise, HSPs and proteostasis pathways have been long linked to chronic airway disease pathogenesis, with elevated levels of eHSP70 previously found in plasma and sputum of patients with COPD, asthma, and asthma-COPD overlap (ACO) and positively correlated with disease severity (32–38). The highly sequence-similar HSC70 has also been found in BALF-derived EVs after olive pollen induced allergic airway inflammation, and these eHSC70 enriched EVs could induce tolerance and protection against the same antigen in naive mice (39). How changes in EV secretion, cargo, and associated proteins such as eHSP70 and eHSC70 contributes to airway disease pathogenesis remains to be shown.

Here we sought to unravel the mechanism of IL-33^Δ34^ unconventional secretion from airway epithelial cells by exploring how cytoplasmic forms of IL-33 are recruited to nSMase2^+^ organelles. Our results demonstrate that HSP70 interacts directly with IL-33^Δ34^ and traffics with the cytokine to facilitate its recruitment for unconventional protein secretion. Once outside of the cell, eHSP70 further appears to stabilize IL-33 by decreasing oxidation/degradation, resulting in enhanced receptor signaling. This chaperoning effect, both inside and outside the cell, has the potential to augment IL-33–driven signaling in chronic airway disease. In the broader context of proteostasis pathways in airway disease, we found significant changes in not only HSP70 but also in HSP90 and the Chaperonin Containing TCP-1 (CCT) complex in lung tissue and bronchial lavage EVs, providing evidence for global dysregulation of cellular proteostasis pathways in COPD.

## Results

### IL-33^Δ34^ interacts with HSP70.

**Figure 1:**
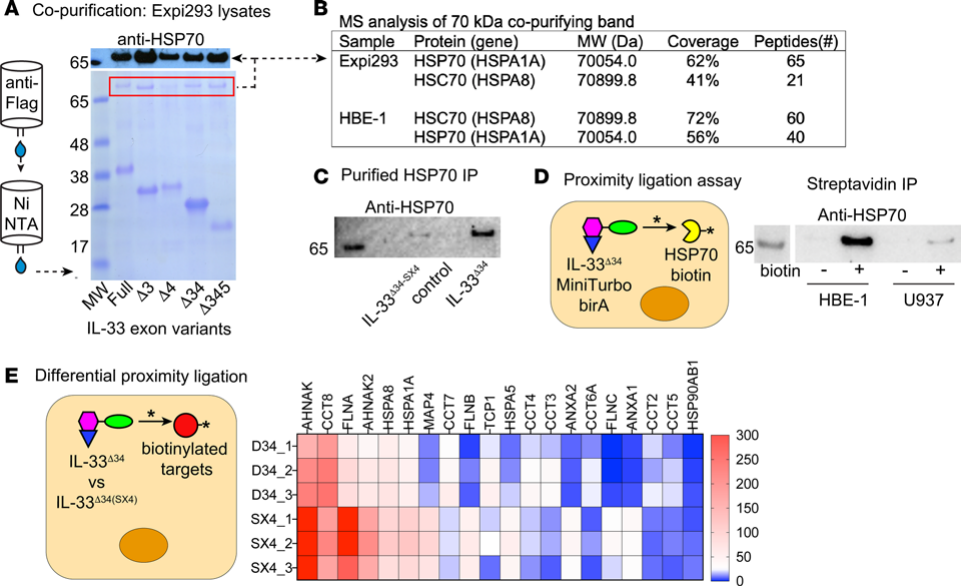
HSP70 interacts with IL-33 in human airway epithelial cells. (**A**) SDS-PAGE gel demonstrating copurification of a high–molecular weight band (>65 kDa, MW markers as indicated) that copurified with all IL-33 isoforms on tandem anti-FLAG, NiNTA affinity purification. (**B**) Mass spectrometry analysis of contaminating band isolated from Expi293 expression cells and HBE-1 airway cell line identified as HSP70 (and HSC70) with coverage and relative number of peptides reported. (**C**) Recombinant purified human IL-33^Δ34^ and HSP70 interact by Co-IP of biotinylated IL-33 protein compared with oxidation resistant 4 cysteine to serine mutant (Cys->Ser x4) mutant IL-33^Δ34-SX4^. (**D**) Intracellular interaction of these proteins verified based on overexpression of miniTurbo IL-33^Δ34^ proximity ligase fusion protein with robust biotinylation of HSP70 in HBE-1 cells but not in U937 cells. (**E**) Differential proximity ligation comparison for IL-33^Δ34^ and IL-33^Δ34-SX4^ miniTurbo fusions in HBE-1 cells. Heatmap of top 20 most abundant proteins and additional heat shock intermediates of relevance. Peptide number indicated by color, with replicates 1–3 as shown.

Previous studies have demonstrated the alternative splicing of IL-33, lacking exons 3, 4, and 5 either individually or in combination, in diseased human airway cells and lung tissue (12, 40). We expressed these variants in mammalian Expi293 cells with dual FLAG and 6xHis tags and performed tandem affinity purification, which revealed a coeluting band of high molecular weight for all IL-33 isoforms (Figure 1A). We identified this coeluting band as containing HSP70 and HSC70 by mass spectrometry analysis (Figure 1B), with ratio depending on cell type analyzed, and this band was immunoreactive to anti-HSP70 by Western blot (Figure 1A). We validated this interaction by coimmunoprecipitation using purified recombinant biotinylated IL-33^Δ34^ and HSP70 expressed in *E. coli* (Figure 1C). Interestingly, there is reduced HSP70 interaction with an oxidation resistant form of IL-33^Δ34^ (IL-33^Δ34-SX4^), in which all 4 surface-exposed cysteine residues in the C-terminal cytokine domain are mutated to serine; SX4 indicates serine × 4) (Supplemental Figure 1F; supplemental material available online with this article; https://doi.org/10.1172/jci.insight.193640DS1) (41), is used as the bait. This indicates that HSP70 interaction is influenced by these C-terminal cysteine residues.

We also demonstrated coordinated intracellular trafficking of IL-33^Δ34^ and HSP70 using the miniTurbo system. The miniTurbo biotin ligase has a 10–15 nm labeling radius and rapidly biotinylates proteins in proximity upon addition of exogenous biotin (42, 43). When the IL-33^Δ34^–miniTurbo fusion construct is expressed in HBE-1 cells, after biotin treatment, HSP70 signal was found to be abundant in streptavidin-captured labeled protein fraction by Western blot (Figure 1D). This appeared to be cell type specific, as the same fusion construct resulted in minimal HSP70 biotinylation using a U937 human monocytic cell line that also exhibits abundant HSP70 expression (Figure 1D).

Other IL-33^Δ34^–labeled proteins from the miniTurbo experiment were identified by mass spectrometry proteomics (Figure 1E and Supplemental Table 4). Because the oxidation resistant IL-33^Δ34-SX4^ demonstrated reduced HSP70 labeling in HBE-1 cells, we performed comparative proximity ligation analysis on these miniTurbo fused IL-33 variants (Figure 1E). The IL-33^Δ34^ interactome revealed differential labeling for several heat shock and proteostasis intermediates, examples include HSP70/*HSPA1A*, HSC70/*HSPA8*, and CCT complex subunits CCT2, -3, -4, -5, -7, and -8 (17). These findings indicate that cytoplasmic IL-33 protein is closely linked to proteostasis pathways in airway cells.

### HSP70 facilitates IL-33^Δ34^ secretion.

**Figure 2:**
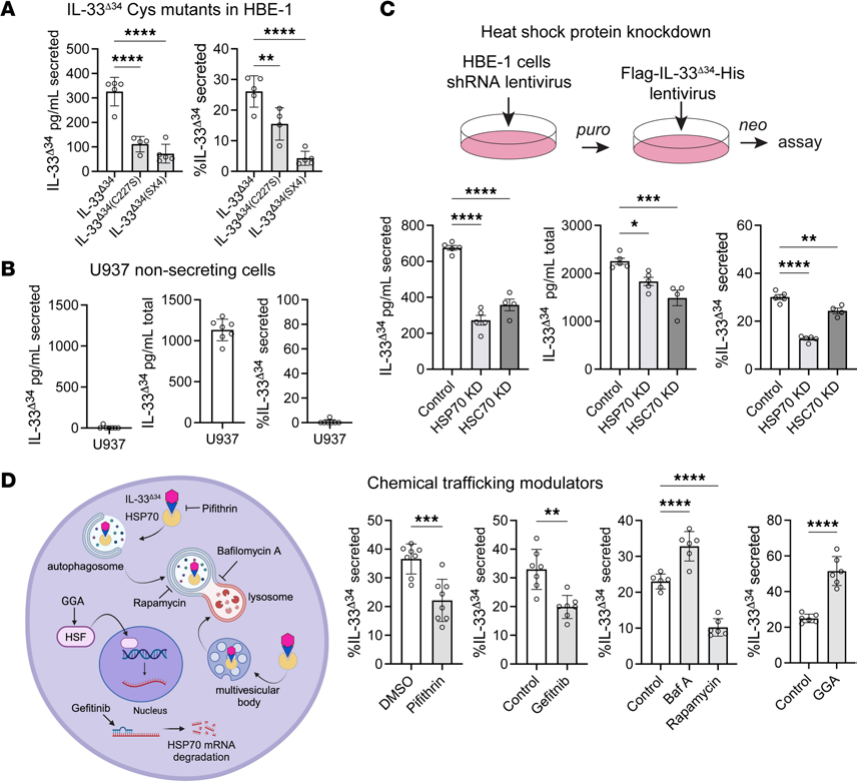
HSP70 facilitates IL-33 secretion. (**A**) HBE-1 cell secretion by ELISA for lentiviral-expressed IL-33^Δ34^, IL-33^Δ34-SX4^, and IL-33^Δ34-C228S^ proteins represented as pg/mL secreted into supernatant and secretion efficiency normalized for total protein expression level (*n* = 5). (**B**) ELISA IL-33^Δ34^ secretion assay in U937 cells that demonstrate no detectable secretion despite robust intracellular expression in the ng/mL range (*n* = 8). (**C**) HBE-1 airway cells were cotransduced with lentiviruses expressing shRNA targeting HSP70 or HSC70 and dual selected for ELISA secretion assay (HSP70 KD, *n* = 5; HSC70 KD, *n* = 4). (**D**) Schematic of HSP70 and vesicular trafficking inhibitors. ELISA secretion assay performed with HSP70 inhibitors: Pifithrin-μ (10 µM) (*n* = 8), Gefitnib (20 µM) (*n* = 7), autophagosome acidification inhibitor bafilomycin A (Baf A, 100 nM), autophagy activator rapamycin (100 nM) (*n* = 6), and HSP70 activator Geranylgeranyl acetone (GGA, 20 µM) (*n* = 6). Inhibitors were preincubated on cells prior to secretion assay. Data are shown as with mean ± SEM. One-way ANOVA (**A**–**C**) and *t* test (**D**). *P < 0.05, **P < 0.01, ***P < 0.001, ****P < 0.0001. **A**–**D** are representative of 3 experiments.

The IL-33^Δ34^ variant is tonically secreted from primary airway basal cells and cell lines without need for stimulus, and this secretion is blocked by the compound GW4869 (12). We show that the oxidation resistant IL-33^Δ34-SX4^ mutant exhibited decreased HSP70 interaction in HBE-1 cells by coimmunoprecipitation (Figure 1C). When we examined secretion, we observed the SX4 mutant form was not secreted into the conditioned media (Figure 2A) and was retained within the cell lysate (Supplemental Figure 1E). Additional single and double-cysteine mutation analyses revealed that this effect was most sensitive to C227 (Supplemental Figure 1E), suggesting the effect may be dependent on this single cystein rather than the oxidation state of the cytokine.

We had previously observed that the HMC-1.2 mast cell line could not tonically secrete IL-33^Δ34^ (12), and we show that U937 cells exhibited reduced HSP70 labeling by miniTurbo (Figure 1D). We therefore examined U937 cells for IL-33^Δ34^ secretion and found these cells were also not capable of tonically secreting IL-33^Δ34^, which was retained within the cellular compartment (Figure 2B). Together, these results support a model in which tonic IL-33^Δ34^ secretion occurs in a cell type–specific manner that is strongly influenced by the presence of C-terminal IL-33 cysteine residues (particularly C227) and involves HSP70.

We therefore hypothesized that HSP70 tethers IL-33 to unconventional secretory organelles for secretion through interactions with the C-terminal cytokine domain. To determine if HSP70 interaction is necessary for IL-33^Δ34^ secretion, we performed RNA interference of HSP70 and HSC70 in HBE-1 cells (Figure 2C). We were able to demonstrate efficient knockdown HSP70 mRNA expression, which is abundant in HBE-1 cells at baseline (Supplemental Figure 1A), and observed a modest decrease in IL-33^Δ34^ secretion when normalized to total IL-33^Δ34^ cellular levels. The effect was less pronounced for HSC70 knockdown, suggesting this chaperone may be less efficient in recruitment. We attributed the modest effect to high baseline expression of both HSP70 and HSC70, which is almost 2 orders of magnitude greater than overexpressed IL-33^Δ34^ in our HBE-1 cells (Supplemental Figure 2D). We therefore complemented this approach with chemical blockade targeting the HSP70 axis using Pifithrin-μ and Gefitinib, inhibitors of HSP70 substrate binding and mRNA translation, respectively (44, 45). We found that both chemical inhibitors decreased IL-33^Δ34^ secretion in our assay, supporting the RNA interference results (Figure 2D and Supplemental Figure 1B).

HSP70 has also been implicated in nonclassical secretion mechanisms that involve secretory autophagy or the EV biogenesis through chaperone-mediated autophagy (CMA) or endosomal microautophagy (46, 47). Using inhibitors of these systems, we saw a significant increase in IL-33^Δ34^ release with bafilomycin A (Baf A) treatment, which inhibits autophagy and augments EV release (48). We observed a corresponding decrease in IL-33^Δ34^ secretion with rapamycin, which promotes autophagy and can decrease EV release (49) (Figure 2D and Supplemental Figure 1C). Treating cells with the HSP70 inducer Geranyl geranylacetone (GGA) (45) also results in more IL-33^Δ34^ secreted (Figure 2D and Supplemental Figure 1D). These inhibitors targeting alternative secretory pathways support our model for HSP70-mediated recruitment of IL-33^Δ34^ to EV biogenesis pathways.

Regarding other forms of IL-33, particularly the nuclear-sequestered full-length form, we previously demonstrated that overexpressed full-length IL-33 (IL-33^full^) is not secreted from airway cells (12). To address the role of HSP70 in secretion of other IL-33 isoforms, we show that HSP70 can interact with all other reported IL-33 isoforms containing the C-terminal cytokine domain (Figure 1A). When nuclear IL-33^full^ trafficking is inhibited using importazole or ivermectin (50), we observe tonic cytokine secretion from HBE-1 cells (Supplemental Figure 1G), indicating that cytoplasmic localization is necessary and sufficient for tonic secretion. Together, these data support our model in which cytoplasmic localization of IL-33 facilitates exposure to HSP70 as a chaperone mediating recruitment of IL-33 to EV secretion pathways.

### IL-33^Δ34^ can bind phosphatidylserine.

**Figure 3:**
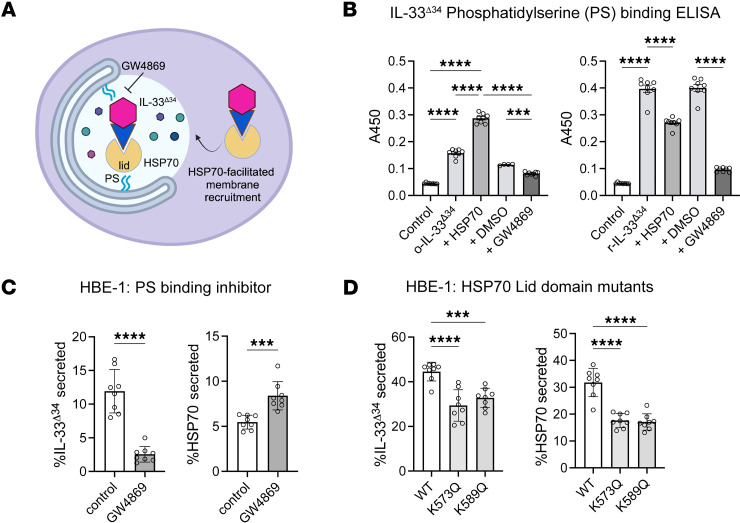
IL-33 interacts directly with phospholipids. (**A**) Schematic of model for phosphatidylserine (PS) cooperative recruitment of IL-33^Δ34^ and HSP70 to a secretory vesicular compartment. (**B**) PS binding ELISA performed using reduced (r-IL-33^Δ34^) and oxidized (o-IL-33^Δ34^) cytokine (100 ng/mL) ± HSP70 (300 ng/mL), GW4869 (20 mM), or DMSO vehicle control. (**C**) Secretion ELISA data for IL-33^Δ34^ and HSP70 under conditions of GW4869 treatment. (**D**) Secretion ELISA data for IL-33^Δ34^ under conditions of lentiviral overexpressed HSP70 LID domain mutants K573Q and K583Q and WT control. Data are shown as mean ± SEM. One-way ANOVA (**A**–**D**). *P < 0.05, **P < 0.01, ***P < 0.001, ****P < 0.0001. For **A**–**D**, *n* = 8 and are representative of 3 experiments.

nSMase2 binding to phosphatidylserine (PS) induces conformational changes that activate the catalytic domain, and GW4869 inhibits nSMase2 by competitively binding PS (51). Given our previously observed blockade of IL-33^Δ34^ secretion using GW4869 (12), we employed a lipid-binding ELISA to test whether the compound could also influence direct cytokine interactions with lipids (Figure 3A). We found that IL-33^Δ34^ directly binds PS preferentially compared to phosphatidylcholine (PC), and this interaction was significantly abrogated by GW4869 (Figure 3B and Supplemental Figure 2B). We also observed that the nonsecreted IL-33^Δ34-SX4^ mutant has significantly reduced ability to bind PS (Supplemental Figure 2A) compared with IL-33^Δ34^. We further tested whether oxidation of the 4 exposed cysteines, which causes a conformational change that disrupts receptor binding (41), could affect lipid binding through examination of the oxidized and reduced IL-33^Δ34^ (o-IL-33^Δ34^ and r-IL-33^Δ34^, respectively) form. We indeed found that oxidation also decreased IL-33^Δ34^ direct binding to PS (Figure 3B). Furthermore, coincubating with HSP70 demonstrated that the chaperone was able to partially rescue PS binding of the oxidized cytokine. Together, these results suggest that IL-33^Δ34^ and HSP70 recruitment to PS membranes may be cooperative and may preferentially occur for the reduced form of the cytokine harboring native cysteines in the C-terminal domain.

The chaperones HSP70 and HSC70 are both able to directly bind PS via electrostatic interactions arising from a cluster of lysine residues in the C terminus of the proteins (52, 53). To test the effect of PS-binding inhibition, we examined cellular secretion of eHSP70 with GW4869 treatment (Figure 3C and Supplemental Figure 2C). In contrast to IL-33^Δ34^, we found that GW4869 does not influence eHSP70 secretion, possibly due to specificity of GW4869 for certain lipid-binding proteins, or due to inadequate dosing, given that total endogenous HSP70 protein is at a much higher concentration than IL-33^Δ34^ in the cellular assay (Supplemental Figure 2D).

Prior structural studies have identified specific lysine residues in the HSC70 LID domain (53) that can disrupt PS binding when mutated. We therefore utilized a dominant-negative approach to investigate the effect on IL-33^Δ34^ secretion by coexpressing 2 PS binding-deficient HSP70 mutants K573Q and K589Q in HBE-1 cells (Figure 3D and Supplemental Figure 2E). Both HSP70 dominant negative mutants decreased secretion for both IL-33^Δ34^ and eHSP70 in conditioned media compared with the control HSP70 WT condition. These results, in addition to PS binding assays, support a model in which HSP70 and IL-33 are recruited to vesicular secretion pathways through interactions with phospholipids.

### HSP70 differentially modulates IL-33 binding and signaling through receptors.

**Figure 4:**
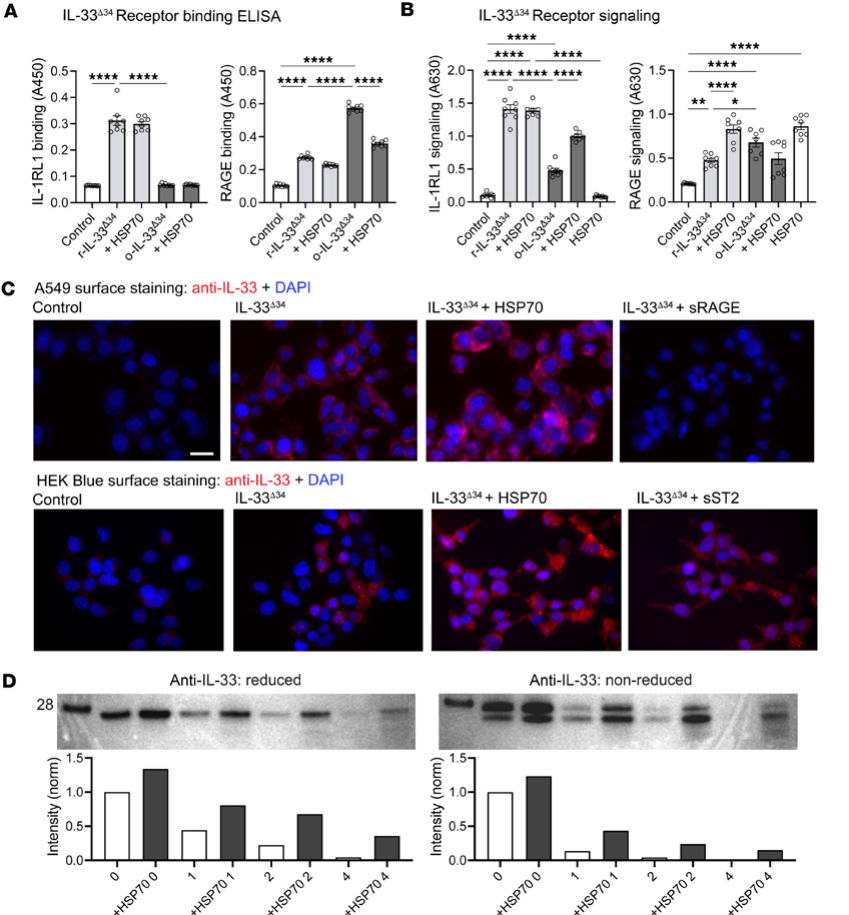
HSP70 preserves oxidized IL-33 receptor signaling. (**A**) Receptor-binding ELISA for IL1RL1 and RAGE binding to reduced (r-IL-33^Δ34^) and oxidized (o-IL-33^Δ34^) cytokine (100 ng/mL) ± HSP70 (300 ng/mL) and with HSP70 alone (known ligand for RAGE, not IL1RL1) (*n* = 8). (**B**) Cellular signaling assays performed under same conditions as in **A** using HEK293T (HEK-Blue, overexpressed IL1RL1/IL1RAP complex and AP-1/NF-κB reporter) or A549 Dual (endogenous RAGE, NF-κB reporter) cell lines with development of secreted alkaline phosphatase (SEAP) as readout (*n* = 8). (**C**) Immunofluorescence staining of A549 Dual and HEK-Blue cells demonstrating IL-33^Δ34^ protein binding (red) to the cell surface ± HSP70 or soluble forms of corresponding receptors RAGE and IL1RL1. DAPI nuclear counterstain (blue). Imaging performed at 60× magnification. Scale bar: 20 mm. (**D**) IL-33^Δ34^ was diluted in media (1 mg/mL) ± HSP70 (10 mg/mL) and aliquots were taken at 0-, 1-, 2-, and 4-hour time points for reduced and nonreduced immunoblots. Quantification of band intensity is below gel images with corresponding lane conditions. Data are shown as mean ± SEM. One-way ANOVA (**A**). *P < 0.05, **P < 0.01, ***P < 0.001, ****P < 0.0001. **A** and **B** are representative of 3 experiments.

Because HSP70 is also tonically secreted from cells (54, 55), we next asked whether this association would influence IL-33^Δ34^ biology outside of the cell. We observed that HSP70 does not compete for r-IL-33^Δ34^ binding to its cognate receptor IL1RL1 (also known as ST2) by receptor binding ELISA (Figure 4A). HSP70 also did not reduce IL1RL1/IL1RAP signaling, measured by secreted embryonic alkaline phosphatase (SEAP) expression in HEK-Blue IL1RL1 signaling reporter cells (Figure 4B). Notably, o-IL-33^Δ34^ was unable to bind IL1RL1, but addition of HSP70 during the oxidation step did partially rescue IL1RL1/ST2 signaling of the oxidized form of the cytokine, suggesting that HSP70 interaction may increase cytokine potency in the extracellular milieu (Figure 4B).

o-IL-33 has been reported to bind and signal through a noncanonical receptor complex of RAGE and EGFR (56), and HSP70 is also a reported RAGE ligand (57). We therefore tested whether HSP70 and o-IL-33^Δ34^ could compete for binding to RAGE and found that, indeed, presence of HSP70 reduced o-IL-33^Δ34^ interaction with RAGE by ELISA (Figure 4A). Likewise, the addition of HSP70 to o-IL-33^Δ34^ reduced signaling through RAGE measured using an NF-κB–driven SEAP reporter assay in A549 cells (Figure 4B), which do not express any detectable IL1RL1 receptor (Supplemental Figure 2F) but do express RAGE (56, 57). HSP70 alone proved to exhibit the most abundant signaling in the A549 RAGE reporter assay but had no effect on signaling in the HEK-Blue IL1RL1 reporter line. These findings support a model whereby eHSP70 can interact with both oxidized and reduced forms of IL-33, with the potential to affect signaling through both canonical and alternative receptors.

To complement ELISA-based receptor binding and SEAP reporter assays, we tested IL-33^Δ34^ binding to cell surface receptors on A549 and HEK-Blue cell lines by immunostaining. We observed an increase in IL-33–specific fluorescence signal in the presence of HSP70 for both cell lines, and this signal was relatively decreased by coincubating with soluble RAGE (sRAGE) or soluble IL1RL1 (sST2), respectively (Figure 4C). This effect was more pronounced for RAGE, likely due to relative higher cytokine binding affinity of overexpressed IL1RL1/IL1RAP complex on the HEK-Blue reporter cells.

These findings led us to test whether HSP70 could stabilize IL-33^Δ34^ in the extracellular environment. Indeed, we found that coincubating HSP70 with IL-33^Δ34^ appears to protect cytokine from both oxidation (duplicate band below the expected 28 kDa band) (41) and degradation (increased laddering and loss of total signal) by Western blot for IL-33^Δ34^ incubated in serum-free culture media with or without HSP70 at 37°C for 4 hours (Figure 4D). Together, these results implicate HSP70 in stabilization of IL-33 upon cellular secretion and suggest that this chaperone can differentially modulate cytokine binding to canonical IL1RL1 and alternative RAGE receptors.

### Heat shock transcripts are modulated in airway disease.

**Figure 5:**
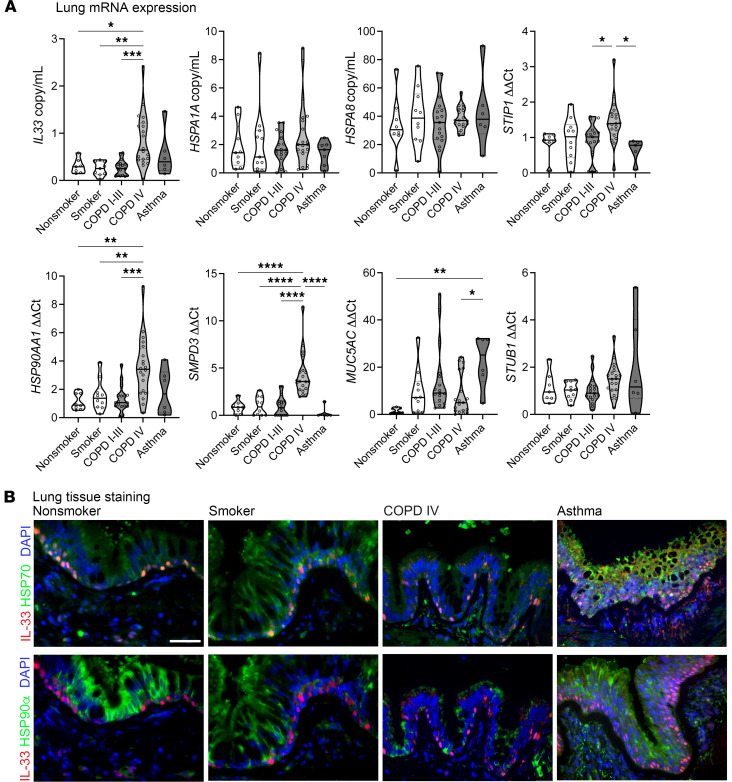
Heat shock intermediates and IL-33 expression in airway disease tissue. (**A**) Human lung tissue specimens were stratified based on GOLD classification and smoking status for nondiseased specimens (Supplemental Table 1). Transcript expression was measured by gene-specific qPCR for nonsmokers (*n* = 7), smokers (*n* = 11) COPD I–III (*n* = 17), COPD IV (*n* = 20), and severe asthma (*n =* 6) lung tissue specimens represented as copy/mL based on plasmid standard or fold-change by ΔΔCt method as shown, normalized to GAPDH. (**B**) Immunofluorescence staining for representative human lung tissue specimens across COPD disease severity and for asthma specimens. Staining for IL-33 (red) and HSP70 or HSP90 (green) as indicated; DAPI counterstain (blue), imaging performed at 40× magnification. Scale bar: 50 mm. Data are shown as mean ± SEM. One-way ANOVA (**A**). *P < 0.05, **P < 0.01, ***P < 0.001, ****P < 0.0001.

We have previously shown that *IL33^full^* and *IL33*^Δ34^ mRNA expression is upregulated in COPD lung tissue (12, 58). Here, we analyzed a cohort of human specimens that represent a spectrum of airway disease severity. We collected and characterized specimens that include nonsmokers, smokers, patients with mild-moderate COPD, and patients with severe COPD as well as those with severe asthma (Supplemental Table 1), and we measured expression levels of several heat shock and proteostasis intermediates by quantitative PCR (qPCR) (Figure 5A and Supplemental Figure 3A). We found, expectedly, that *IL33*, *SPMD3* (nSMase2), and *MUC5AC* were increased with smoking and progression of airway disease and correlated with increased *IL33* expression (Supplemental Figure 3B). Interestingly, *SMPD3* expression was prominently and specifically increased in severe COPD and not modulated in the (smaller) asthma cohort. And while we did not observe statistically significant changes in *HSPA1A* or *HSPA8* transcript levels, other stress-induced intermediates — including the inducible form of HSP90 (HSP90α/*HSP90AA1*) and the heat shock cochaperone *STIP1* (59) — were upregulated in severe COPD (Figure 5A). Other vesicular trafficking intermediates tested were not differentially expressed, although the proteostasis moderator p62/*SQSTM1* exhibited a trend toward decreased expression in severe disease (Supplemental Figure 3A). These expression data support a concept of altered expression for proteostasis intermediates with airway disease severity.

### HSPs are altered in airway disease.

To complement transcript level expression data, we also investigated protein expression patterns for heat shock intermediates in airway disease tissue. We found that HSP70 showed diffuse localization throughout the epithelium but was present and relatively concentrated within IL-33–expressing basal cells (Figure 5B). In contrast, HSP90α protein exhibited intense immunostaining in ciliated airway cells, with relative exclusion from IL-33–expressing airway basal cells. Comparison across disease states demonstrates relative decline in HSP70 airway staining in severe COPD specimens, with diffuse, patchy staining in areas of airway basal hyperplasia in severe asthma. By comparison, HSP90α protein staining was also decreased in severe COPD airways, with a relative paucity of staining observed in asthma as well, which appears to reflect the abundant mucus metaplasia in both severe COPD and severe asthma. These results indicate that intracellular protein levels of heat shock intermediates appear to be uncoupled from transcript expression in airway disease, and both are affected by COPD disease severity and airway disease phenotype.

**Figure 6:**
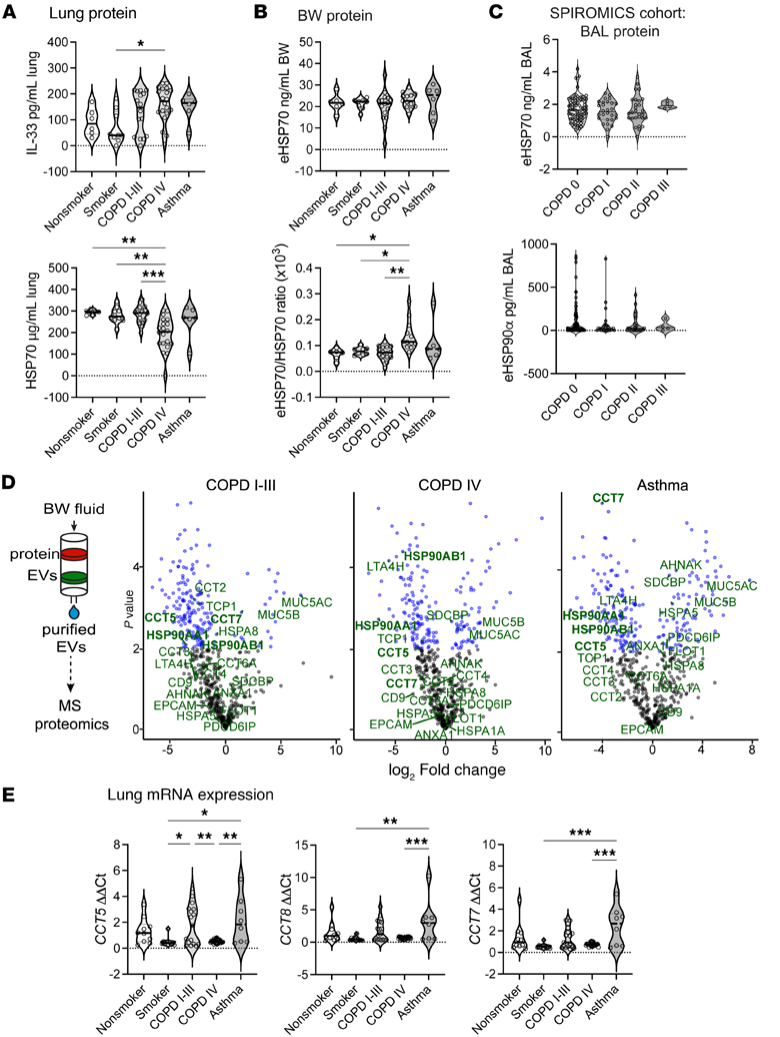
Heat shock intermediates and IL-33 are present in BALF, and EV contents is altered in disease. (**A** and **B**) Protein levels for IL-33 and HSP70 in lung tissue and bronchial wash (BW) fluid measured by ELISA for nonsmokers (*n =* 6), smokers (*n* = 10), COPD I–III (*n* = 16), COPD IV (*n* = 20, BAL *n* = 13), and severe asthma (*n* = 6) specimens. (**C**) HSP70 and HSP90 protein levels measured by ELISA in SPIROMICS BAL specimens for COPD 0 (*n =* 91), COPD I (*n =* 28), COPD II (*n =* 29), and COPD III (*n =* 4) sample groups. (**D**) Schematic for EV isolation by size exclusion chromatography from BW and volcano plots for differential protein expression in COPD IV, COPD I–III, and asthma specimens compared with nonsmoker control. Proteins annotated are associated with heat shock or proteostasis systems and were biotinylated by IL-33 in miniTurbo experiment (Figure 1E). BW EV proteome demonstrated for HSP70, HSC70, and HSP90. Each sample was analyzed in triplicate and plotted in aggregate. (**E**) Transcript expression of molecular chaperonin TCP1 subunits *CCT5*, *CCT7*, and *CCT8* in lung tissue displayed as fold-change by ΔΔCt method with normalization to *GAPDH* for nonsmokers (*n* = 10), smokers (*n* = 11) COPD I–III (*n* = 17), COPD IV (*n* = 22), and severe asthma (*n* = 8) groups. Data are shown as mean ± SEM. One-way ANOVA (A-C, E) and *t* test (**D**). *P < 0.05, **P < 0.01, ***P < 0.001, ****P < 0.0001.

We also measured total protein concentrations of IL-33, HSP70, HSP90α, soluble ST2 and RAGE in diseased tissue lysates and bronchial wash (BW) fluid. As anticipated, IL-33 tissue protein levels trended upward with disease severity, while HSP70 amounts decreased in an inverse manner (Figure 6A). Despite decreases in tissue HSP70 levels, BW eHSP70 concentrations remained relatively constant (Figure 6B), though the ratio of BW/total lung HSP70 (eHSP70/total HSP70), as an indirect measure of extracellular versus intracellular fractions, was significantly increased in severe COPD. A similar result was observed for eHSP70 concentrations measured in BAL from the subpopulations and intermediate outcomes in COPD Study (SPIROMICS) cohort (60), though the absolute concentration of protein was 10-fold lower, likely a dilutional effect due to sampling technique (Figure 6C). Notably, severe COPD was under-represented among SPIROMICS BAL samples, which did not allow for direct comparison of severe disease across cohorts. For HSP90α, lung tissue and BW specimens exhibited a very broad range of concentrations, which decreased in BW fluid for all disease states compared with nonsmokers (Supplemental Figure 4A). A similar nonsignificant decreasing trend was observed in the SPIROMICS BAL specimens (Figure 6C). These trends likely reflect the increased mucus metaplasia as a function of disease severity, as we observed for tissue immunostaining. Additionally, low extracellular HSP90α (eHSP90α) protein signal could be related to preferential incorporation of HSP90 into (<50 nm diameter) nonmembranous exomeres/supermeres (61) rather than small EVs as for HSP70 (26, 62), which may be more difficult to detect by ELISA. Measurement of sST2 and sRAGE in SPIROMICS BAL did not demonstrate a significant change across disease severity (Supplemental Figure 4B) but did show a nonsignificant downtrend for sRAGE, which likely reflects progressive emphysema and loss of type-1 pneumocytes. The lack of effect as a function of disease severity may be due to underrepresentation of severe disease in this cohort.

### Differential EV-mediated secretion of HSPs in airway disease.

The link between increases in nSMase2 activity and HSPs is well established in the field of EV biology, and both are linked to EVs of endolysosomal origin (26). We have previously shown that IL-33^Δ34^ associates with purified EVs isolated from lung lavage fluid (12). Therefore, our next step was to isolate BW (BW EVs) from specimens across the spectrum of COPD disease severity to examine the secreted EV proteome for comparison to total tissue and bronchial lavage levels. We isolated EVs using size exclusion chromatography (SEC), as per our established protocols (12), and validated that our preparation contained tetraspanin (CD9, CD81, CD63) marker positive particles with the SP-IRIS imaging (Supplemental Figure 4C). The purified BW-derived EVs from nonsmoker, COPD I–III, COPD IV, and asthma specimens were then subjected to differential mass spectrometry proteomics compared with nonsmoker control (Figure 6D and Supplemental Table 5). As anticipated, we observed increased MUC5AC and MUC5B mucin peptides associated with EVs isolated from COPD and asthma BW specimens. HSP70 and HSC70 were detected in EVs from in all samples but were not found differentially expressed between samples. We did not detect IL-33; however, mass spectrometry–based detection of this cytokine from biospecimens has not been successful in our hands. Interestingly, both stress inducible HSP90α/*HSP90AA1* and constitutive HSP90β/*HSP90AB1* were decreased in BW EVs in concert with downtrending protein levels in BW and BALF and reduced immunostaining in airway disease. Multiple intermediates of leukotriene metabolism (LT4AH, ALOX15), relevant in pathogenesis of airway disease and known to be EV associated (27), were found to be decreased in BW EVs from patients with COPD and asthma. We also found multiple subunit proteins from the cytosolic CCT/TCP1 ring complex (TRiC), including CCT2, CCT4, CCT5, CCT7, CCT8 (17, 63), were decreased in airway disease BW EVs. These chaperonins were of great interest because our miniTurbo proximity assays in Figure 1E indicated CCT complex proteins were labeled by IL-33, implicating this complex in cytokine folding, cellular trafficking (64), and/or retention. Accordingly, we measured mRNA expression levels for the *CCT5*, *CCT8*, and *CCT7* subunits in airway disease lung tissue and found that they were downregulated in smokers and those with severe COPD (Figure 6E). Together these data indicate a potential role for the CCT complex in unconventional secretion pathways that influence IL-33 biology and highlight yet another component of cellular proteostasis machinery that is dysregulated in airway disease.

## Discussion

HSPs and proteostasis pathways have long been linked to chronic airway disease pathogenesis. It has been postulated that HSP70 has antiapoptotic functions and, as part of the cellular stress response, brings cells back into homeostasis (65). While the vital intracellular functions of this chaperone have been highly studied, a role for eHSP70 is less well defined, and whether the secreted chaperone functions in a pro- or antiinflammatory manner in human airway disease is currently debated (55). For example, increased expression levels of *HSPA1A* mRNA have been observed in COPD lung tissue and mucosal scrapings from patients with allergic rhinitis (36), which is positively correlated with disease severity and smoking status (34). eHSP70 concentration was also found to increase with disease severity in the sputum and serum of patients with asthma (37, 66), and in plasma (35) and sputum of patients with COPD (33). Increased protein concentrations of eHSP70 were also found in sputum and serum or plasma of patients with asthma, COPD, and ACO (32), and these increased concentrations were associated with disease severity, negatively correlated with reported lung function parameters (32, 33, 37), and positively correlated with FeNO concentration (36). These associations likely stem from the role of HSPs in response to cellular stress, toxin-mediated tissue damage, and hypoxia, which are common in the diseased airway tissue environment.

To address a mechanistic role for HSP70 in the context of airway inflammation, several preclinical models have been employed, and both pro- and antiinflammatory effects of HSP70 have been reported (66–68). For example, in an OVA airway inflammation model, exogenous oropharyngeal administration of murine HSP70 after OVA challenge attenuated type 2 cytokine production and eosinophilia (66). Conversely, HSP70 (*HSPA1A/B*) global KO mice challenged intratracheally with Schistosoma mansoni soluble egg antigens (SEA) demonstrated reduction in Th2 cytokine production, goblet cell hyperplasia, and airway inflammation (67), which was attributed to decreased response of HSP70-deficient hematopoietic cells.

Our work here suggests that proteostatic dysregulation can lead to increased externalization of HSP70, its cargo, and cargo destined for degradation that may contribute to airway disease pathology in the human system. We link intracellular HSP70 to recruitment of IL-33 for noncanonical secretion from airway cells and eHSP70 to potential stabilization of the cytokine outside of the cell. Additional work will be necessary to fully characterize the protective effect of eHSP70 with respect to IL-33 oxidation and degradation and the relative contributions of this chaperone to cellular effects mediated through RAGE signaling in airway disease.

Our results identify a previously unrecognized function for HSPs in facilitating the secretion and function of a mislocalized proinflammatory cytokine. We have further demonstrated that this function requires the presence of cysteine residues 208, 227, 232, and 259 on IL-33, raising the possibility that covalent HSP70 adducts could contribute to this stabilization function. Such an adduct may hold IL-33 in an active conformation in an oxidating environment to maintain potency and promote airway disease. Given that IL-33 does not contain the canonical KFERQ binding motif typical of HSP clients (69), this may be highly relevant to the mechanism of interaction, which will require structural and biophysical characterization in future studies.

There are multiple layers of regulation in the HSP system that help route HSP70/HSC70 and cargo for degradation or secretion depending on cell nutrient status, through changes in HSP posttranslational modifications (70), or association with other cochaperones (59). In a recent study, deficiency in cochaperone BAG6, which can shunt HSC70 and associated clients to the lysosome or the endosome depending on nutrient availability (71), facilitated pancreatic tumor release of EV-associated IL-33 (72).

Other nonclassically secreted cytokines exhibit analogous secretory behavior to IL-33 in vitro, including FGF-1 and mature IL-1a (m-IL-1a) (73). Upon cellular stress, both have been shown to associate with PS and other PS-binding and EV-associated proteins (like ANXA1/2) (74), which facilitate secretion. Furthermore, the effect of surface-exposed cysteines on FGF-1 binding to PS and subsequent secretion has been demonstrated (75). Furthermore, like IL-33, IL-1a can be secreted with EVs from stimulated mouse neutrophils, and its release was inhibited with GW4869 treatment (76). Taken into context, our results implicate ER/Golgi-independent unconventional protein secretion pathways (77) in the recruitment of mislocalized proteins and changes in cellular secretome contributing to disease pathogenesis.

In our analysis of airway disease specimens, we observed an increase in *HSP90AA1* transcript with decreased airway immunostaining as a function of disease severity. Prior studies examining HSP90α in plasma of patients with COPD (35) demonstrated an increase with disease severity of modest significance, suggesting that HSP90 levels may not be as sensitive and specific for disease. Our COPD airway staining demonstrated that HSP90α localized to the mucosal surface, which is also observed in the gut mucosa of induced colitis mouse models (78). HSP90 function was found to be required for goblet cell metaplasia in response to IL-13 or IL-17 signaling (79), suggesting that this heat shock family member may be relevant to type 2 cellular responses but may function separately from HSP70 and in distinct cell types.

We have also identified, through a combination of proximity ligation assay and bronchial lavage EV proteomics, a modulatory axis that may be relevant to inflammatory protein secretion through CCT complex proteins (63). The CCT complex is an important part of the molecular chaperone system and is composed of 2 stacked rings, each made of the eight 60 kDa subunits. It is required for folding of approximately 10% of the proteome, especially cytoskeletal components (tubulin, actin) and cell cycle regulators (63). Although the mechanism by which CCT recognizes substrates is still unknown, HSP70 can act as a cofactor by transferring nascent protein chains to the CCT complex (63). This chaperone complex has been implicated in neuropathology due to accumulation of protein aggregates, such as Huntington’s, Parkinson’s, and Alzheimer’s diseases; increased risk for myocardial infarctions; poor cancer survival; and epithelial-mesenchymal transition (EMT) (63). CCT subunits are upregulated in cancer EVs, and CCT4 can serve as a regulator of vesicle trafficking (80). CCT2 can also function as an autophagy receptor by targeting protein aggregates to the autophagosome for degradation (63, 81). The reduction in CCT subunits and HSP90 in COPD and asthma EVs suggests that collective decline in tissue proteostasis mediators may be indicative of chaperone system exhaustion, with a potential role as biomarkers of disease progression or response to treatment. Studies are ongoing to investigate lung cell–specific functions of the CCT complex in airway homeostasis, EV-mediated cellular communication, and the effect of environmental factors on development of chronic disease.

## Methods

### Sex as a biological variable.

This study was conducted using human specimens obtained from patients undergoing lung transplantation and includes male and female patients based on clinical indication.

### Human lung samples and study design.

Clinical samples were obtained from consenting patients at the time of lung transplantation from recipients with COPD (*n* = 20) with very severe disease (Global Initiative for Chronic Obstructive Lung Disease [GOLD] stage IV; Supplemental Table 2) during the period from 2016 to 2025 at Barnes-Jewish Hospital (BJH) (St. Louis, Missouri, USA). Specimens from patients who were nondiseased (nonsmoker *n* = 7 and smoker *n* = 11), with mild-moderate COPD (GOLD stage I–III), and with severe asthma (*n* = 8) were obtained from lungs not suitable for transplantation at BJH (Supplemental Table 1). There were no predetermined inclusion or exclusion criteria beyond criteria for lung transplant candidacy. To analyze tissue staining, gene expression, and protein levels, lung tissue samples were collected and processed for histopathology, RNA, and protein analysis from 4 different lung zones of each specimen and combined for analysis. Equivalent quantities from the 4 lung areas were pooled for RNA and protein analysis to represent a single sample per specimen. Tissue was homogenized in Trizol (Invitrogen) for RNA extraction or minced and lysed in T-PER (Pierce) supplemented with HALT protease inhibitor (Pierce) for protein analysis. Tissue specimens were fixed in 10% neutral buffered formalin (Thermo Fisher Scientific) prior to paraffin embedding and sectioning for histopathology analysis. BW fluid was obtained from explanted lungs by instilling 100 mL of PBS into mainstem bronchi and fluid recovered with passive return and gentle suctioning. BW fluid was centrifuged at 100*g* to pellet cells, and HALT was added to supernatant prior to storage for further analysis. BALF was obtained from the SPIROMICS (60) cohort through NHLBI BioLINCC (https://biolincc.nhlbi.nih.gov/studies/).

### IL-33^Δ34^ and HSP70 Co-IP.

For IL-33^Δ34^ and HSP70 coprecipitation analysis using purified proteins, 5 µg of purified biotinylated IL-33^Δ34^ was bound to Pierce Streptavidin Magnetic Beads for 15 minutes at 4°C in PBS 0.05% Tween20. Beads were washed 3× and incubated with 7.5 µg of HSP70 for 30 minutes at 4°C followed by 3 washes. Proteins were eluted by boiling in 1× SDS PAGE buffer and analyzed by Western blot using Invitrogen iBlot system and anti-HSP70 antibody according to reagent list.

### MiniTurbo proximity ligation assay.

MiniTurbo was cloned as an N-terminal fusion to IL-33^Δ34^ with (GGS)^2^ linker on a pCDH lentivirus vector backbone (VectorBuilder). Lentivirus was prepared as previously reported (12), and HBE-1 cells were transduced and selected for stable expression. Proximity biotinylation was performed as previously described (42). Briefly, cells were switched to media containing 500 µM exogenous biotin and incubated for 2 hours, washed, lysed in 700 µL RIPA lysis buffer, and incubated with 30 µL of strep magnetic beads at 4°C for 1 hour with rocking. Beads were washed 2× with 1 mL RIPA buffer, 1× with 1M KCl, 1× with 0.1M Na_2_CO_3_, 1× with 2M urea 10 mM Tris-HCl (pH 8.0), and 2× with 1 mL RIPA buffer. For trypsin digest and proteomic analysis, biotinylated proteins were eluted in 1× SDS PAGE loading buffer supplemented with 20 mM DTT and 2 mM biotin. Trypsin digest and mass spectrometry were performed according to below.

### Lipid binding ELISA.

Lipid binding ELISA was performed according to previous studies (82). Briefly, Nunc MaxiSorp 96-well plates (Thermo Fisher Scientific) were coated with 5 µg/mL of PS or PC (Avanti Polar Lipids) diluted in methanol, allowed to air dry, and blocked with PBS 1% BSA for 2 hours. Plates were washed 3× with PBS 0.5% Tween. Nonbiotinylated IL-33^Δ34^ (500 ng/mL) ± HSP70 (500 ng/mL) were bound at room temperature for 30 minutes, plates were washed 3×, and bound IL-33^Δ34^ was detected using R&D DuoSet Human IL-33 biotinylated detection antibody following manufacturer protocols and developed using streptavidin HRP (R&D Systems) and TMB peroxidase substrate (SeraCare).

### Quantitative secretion assays.

HBE-1 cells were cultured on collagen-coated tissue culture plates in UNC BEGM media and U937 cells cultured in RPMI/10% FBS/pen-strep (unless otherwise indicated). All secretion assays were performed at 37°C and 5% CO_2_. Media were exchanged to fresh prewarmed media at the beginning of the assay, and plates were incubated for 2 hours. Supernatant was transferred to anti–IL-33 or an HSP-coated ELISA plate, and cells were lysed in MPER (Pierce) supplemented with HALT protease inhibitor (Pierce). Lysates were diluted 1:20 in PBS for HSP70 measurements. For all secretion assays, protein was quantified in supernatant and lysate using R&D commercial kits with total assay protein (supernatant + lysate) and percent secretion (supernatant/[supernatant + lysate] × 100) quantified based on standard curve. Directly measured secreted cytokine quantities (pg/mL) and total cytokine levels (pg/mL) are reported in Supplemental Data for all secretion experimental results reported in main text as percentage secreted.

For chemical inhibition assays, all chemicals were solubilized in DMSO and filter sterilized prior to use. As a control, DMSO was used at the highest concentration required for solubility in the respective assay. Inhibitors were preincubated with cells for 1 hour prior to beginning secretion assay (GW4869, GGA) or 24 hours (Gefitinib, Pifithrin, Rapamycin, Baf A) and maintained in media during the assay. Chemical concentrations used for inhibition assay are as follows: PBS; DMSO vehicle control (2.5%); GW4869 (20 µM), Geranylgeranylacetone (GGA) (20 µM), Pifithrin (10 µM) and Gefitnib (1 µM), Rapamycin (100 nM), and Baf A (100 nM).

### HSP lentiviral knockdown in HBE-1.

HSP70/*HSP1A1* or HSC70/*HSPA8* expression knockdown was carried out using commercially available lentivirus encoding 3–4 shRNA constructs targeting GOI (Santa Cruz Biotechnology Inc.) according to previous studies (12). To summarize, 1 × 10^5^ IFU of virus was combined with TransDux MAX transduction reagent (SBI) and 1 × 10^5^ HBE-1 cells in BEGM media and placed under selection after 24 hours incubation. After recovery from selection, cells were used for experimentation, with an aliquot retained for validation of knockdown by qPCR.

### Bronchial wash EV isolation.

EVs were isolated from BW fluid as per previous and in accordance with MISEV2024 guidelines (12, 83). Briefly, BW was centrifuged at 3,000*g*, concentrated, and fractionated on a qEV 35 (Izon Science) size exclusion column. EV analysis was performed using ExoView R100 imaging and transmission electron microscopy (not shown) or cryo-EM imaging. Isolated EV preps were submitted for mass spectrometry analysis.

### Receptor binding and ELISA.

For ST2 and RAGE binding assays, ELISA plates were coated with 1 mg/mL of commercial soluble ST2 diluted in PBS or with 0.5 mg/mL anti–Human-Fc (BD Biosciences) followed by capture of sRAGE-Fc chimera according to manufacturer instructions; catalog and clone information for all antibodies used is reported in Supplemental Table 3. For sRAGE and ST2 assays, commercial ELISA kits were coated according to manufacturer instructions. Plates were blocked with PBS 1% BSA for 1 hour, and IL-33^Δ34^ was added at 500 ng/mL with or without HSP70 (500 ng/mL). Proteins were diluted in PBS immediately before assay for reduced conditions and incubated in serum-free media at 500 ng/mL with or without HSP70 (500 ng/mL) for 4 hours at 37°C. IL-33^Δ34^ binding was detected using R&D IL-33 detection antibody and developed using streptavidin HRP (R&D Systems) and TMB peroxidase substrate (SeraCare).

### IL-33 receptor cell-surface staining.

For surface staining, cells were plated in 8-well chamber slides and allowed to reach ~70% confluency. IL-33^Δ34^ was diluted in serum-free media to a final concentration of 100 ng/mL. For competition conditions, HSP70 was added at a concentration of 300 ng/mL and sRAGE-Fc chimera or sST2 at a final concentration of 500 ng/mL. Cells were incubated for 15 minutes at 25°C, washed, and fixed with 4% PFA for 5 minutes. Cells were then blocked for 1 hour with 2% BSA/1% Fish Gel in PBS. Surface-bound IL-33 was stained with Nessy-1 (1:1,000) and anti–mouse-AF647 (1:1,000) for A549 staining or polyclonal rabbit anti–IL-33 (1:1,000) before being signal amplified using VectaFluor Excel 594 Amplifier Kit (Vector Labs); see Supplemental Table 3 for antibody details.

### Signaling assays.

HEK-Blue IL-33 reporter cells (Invivogen) were used to assay ST2 signaling, and A549-Dual cells were used to assay RAGE signaling. For signaling, 30,000 cells were seeded per well of a 96-well plate. For receptor signaling, proteins were diluted to 100 ng/mL (IL-33^Δ34^) and 300 ng/mL (HSP70) in serum-free DMEM or Ham’s F-12 media. Cells were switched to serum-free media containing IL-33^Δ34^ ± HSP70 and incubated at 37°C for 2 hours. Cells were then switched back to full media overnight, and SEAP detection was carried out following manufacturer instructions (Invivogen).

### Statistics.

For statistical analysis, 2-tailed Student’s *t* test was used for comparisons between 2 groups, and comparisons with 3 or more groups were analyzed using 1-way ANOVA. For all experiments, *P* < 0.05 was considered statistically significant. Correlation analysis was performed based on Pearson’s coefficient. For all data in which 3 or more independent measurements are reported, representative data are displayed as mean ± SEM.

### Study approvals.

All human studies were conducted with protocols approved by the Washington University IRB, and written informed consent was obtained from study participants.

### Data availability.

Data values from graphs associated with main and supplemental figures is provided in the Supporting Data Values file. All proteins detected for MiniTurbo and BAL EV proteomics experiments corresponding to Figure 1 and Figure 6, respectively, are contained in Miniturbo_Fig1 and Supplemental Tables 4 and 5. Additional relevant data that support the findings of this study not included in this manuscript are available from corresponding author upon request. Restrictions may apply to sharing of human subject data.

## Author contributions

OAO, CEK, HER, GFH, LSC, DFS, MDP, EML, and JAB designed and/or performed the experiments. OAO and JAB prepared figures and wrote the manuscript. JLH, EKK, and DEB contributed to enrollment of human subjects and biobanking efforts.

## Supplementary Material

Supplemental data

Unedited blot and gel images

Supplemental table 4

Supplemental table 5

Supporting data values

## Version 1 06/24/2025

In-Press Preview

## Version 2 08/08/2025

Electronic publication
