# Supplementary material for: HSP70 is a chaperone for IL-33 activity in chronic airway disease
Source: JCI Insight. 2025 Jun 24;10(15):e193640. doi: 10.1172/jci.insight.193640 (PMC12333954; doi:10.1172/jci.insight.193640)
Supplement: Supplemental data [file jciinsight-10-193640-s065.pdf]

SUPPLEMENTARY FIGURES AND METHODS

Figure S1. Associated with Fig. 2.

Figure S2. Associated with Fig. 3 and 4.

Figure S3. Associated with Fig. 5.

Figure S4. Associated with Fig. 6.

Table S1 & S2. Human subject demographics & clinical data.

Table S3. List of reagents associated with Methods.

SUPPLEMENTARY METHODS

Recombinant IL-33 protein expression and purification

Splice variants of IL-33 protein were cloned as per previous (1) into pCDH lentiviral vectors or into pHLSEC (2) for expression in Expi293 system (Invitrogen). For mammalian expression, Flag-IL-33$^{\Delta 34}$-His in pHLSEC was transfected into Expi293 cells using Hype293 (OZ Biosciences) and protein was purified by tandem anti-Flag (GenScript) and NiNTA resins according to protocol. Recombinant protein and co-purified bands were visualized on SDS-PAGE and western blot performed for confirmation of bands. Co-purified HSP70 and HSC70 were confirmed by Mass Spectrometry (methods per MTAC core, see below). Recombinant IL-33$^{\Delta 34}$ for binding experiments was cloned into pET21a (Novagen) with an N-terminal hexahistidine (6XHis) tag, followed by an N-terminal AviTag™ sequence (GLNDIFEAQKIEWHE) and $(GGS)^2$ linker. The IL-33$^{\Delta 34}$ construct was co-transformed with BirA ligase for in-situ biotinylation as needed. HSP70 was cloned with an N-terminal hexahistidine (6XHis) tag into pETDuet vector (Novagen). Mutants were generated using the Agilent QuickChange II XL kit per manufacturer protocol. Constructs were transformed into Rosetta2(DE3) *E. coli*, (Novagen) under antibiotic selection and bacteria were grown in Luria Broth (LB) at 37°C to an OD_{600} of ~0.8 before induction with 0.5mM of IPTG at 20°C overnight.

Protein purification from cell pellets was performed by lysis with sonication in 50 mM Na_2HPO_4 pH 8.0, 300mM NaCl, 5mM imidazole, 10mM β-ME and 10% glycerol with 50 mg/ml Lysozyme (bacterial produced protein), 1 mg DNAse, 1 mM $MgCl_2$, 1mM phenylmethylsulfonylfluoride (PMSF). Following centrifugation at 7,000 x g, supernatants

were passed over NiNTA superflow resin (Qiagen) with increasing imidazole concentration according to manufacturer protocols based on buffer: Na_2HPO_4 pH 8.0, 300 mM NaCl, 10 mM imidazole, 10% glycerol, 5 mM DTT and 10% glycerol supplemented with protease inhibitor cocktail (Sigma). Proteins further purified by size exclusion chromatography (SEC) using a Superdex™ 75 Increase column for IL-33 and a Superdex™ 200 Increase column for HSP70 purifications.

Mass Spectrometry Proteomics

Mass Spectrometry analyses were performed by the Mass Spectrometry Technology Access Center at McDonnell Genome Institute (MTAC@MGI) at Washington University School of Medicine. Briefly, samples were reduced, alkylated, and digested with trypsin according to core facility protocols. Digested peptides were desalted on C18 spin columns and analyzed by mass spectrometry. Data was searched against a Human database using MaxQuant search engine and then performed label-free quantification (LFQ) based on the MS1 peptide intensity.

Extracellular vesicle-derived protein was filtered for >1 unique peptide, which yielded 577 proteins, including several EV marker proteins such as ANXA1, ANXA2, ACTN4, CD81, CD9. Obtained LFQ intensities were Log2 transformed, and data were grouped into groups: MTS15 (asthma), MTS16 (nonsmoker), COPD34 (COPD I-III), and COPD53 (COPD IV). These were filtered to retain proteins with at least 60% intensity values in at least one group which resulted in 565 proteins. Imputation was applied to fill in missing values based on normal distribution, which is recommended for label-free quantification.

Finally, *t*-test was carried out and results visualized by volcano plot to determine significant differences in 6 comparison sets.

MiniTurboID labeled proteins were in-gel digested with trypsin using core facility optimized protocol. Peptides were subject to mass spec analysis and the data searched against a human database. In total, 238 proteins were identified including the presence of several naturally occurring biotin proteins that are used as positive controls for biotinylated enrichment experiments (ACACA, PC, MCCC1, MCCC2, PCCA). Results were visualized using Scaffold 5.0.

SUPPLEMENTARY FIGURES

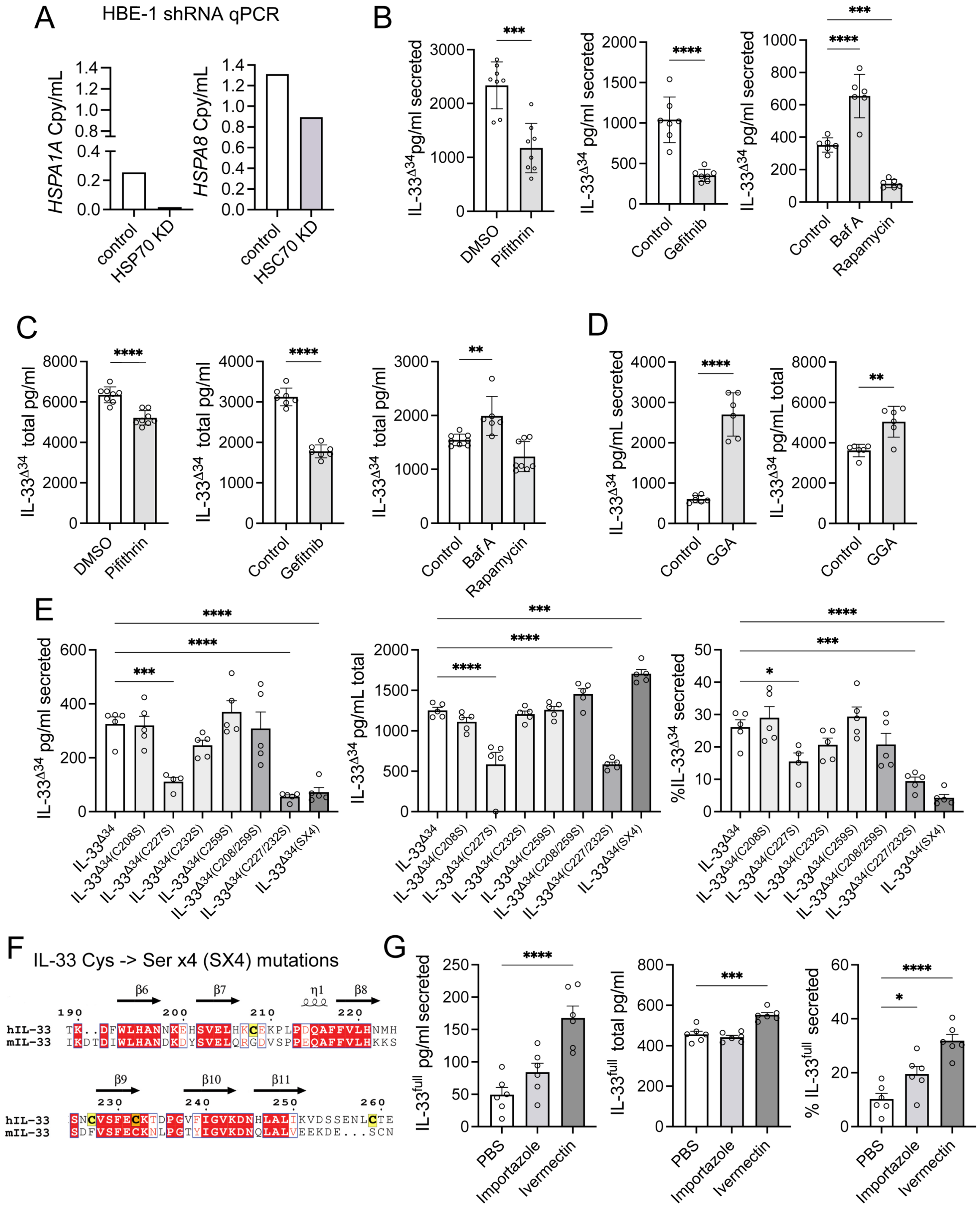

FIGURE S1. Supporting data for Figure 2. A) Validation of shRNA knockdown for HSP70 (*HSPA1A*) and HSC70 (*HSPA8*) in HBE-1 airway cell line (HBE) by qPCR represented as copies/ml (Cpy/ml) based on plasmid standard. B, C, D) Lentiviral-

expressed Flag-IL-$33^{\Delta34}$-His secreted (B) and total protein (C) measured by ELISA from HBE cell supernatant under conditions of inhibitors as shown (Pifithrin (PES) 1 μM, (*n* = 8), Gefitinib 1 μM (*n* = 7), Bafilamycin A (Baf A) 100 nM (*n* = 6), Rapamycin 100 nM (*n* = 8), and (D) GGA 20 μM, (*n* = 6)); control condition indicates PBS treatment unless otherwise indicated (i.e. DMSO). E) Flag-IL-$33^{\Delta34}$-His secretion data under conditions of single or double Cys to Ser mutants as indicated, inclusive of the 4 Cys to Ser mutant (SX4) and wildtype residues C208, C227, C232 and C259 (*n* = 5, for C227S *n* = 4). F) Sequence alignment of human (hIL-33) and mouse (mIL-33) segment of C-terminal domain highlighting the relevant cysteines mutated to serine in E) (bold, yellow); secondary structure of this region in human cytokine indicated above sequence based on PDB ID 4KC3. G) Full-length IL-33 (Flag-IL-33^{full}-His) secretion efficiency in HBE cells under conditions of inhibitors Importazole 20 μM, (*n* = 6) and Ivermectin 10 μM (*n* = 6). Secretion experiments in B, C, D, E and F were repeated 3 times. Data points displayed with mean ± SEM. Statistical analysis: 1-way ANOVA (B, C, E, F) and *t* test (B, C, D). *P*-value limits: **P* < 0.05, ***P* < 0.01, ****P* < 0.001, ****P* < 0.0001.

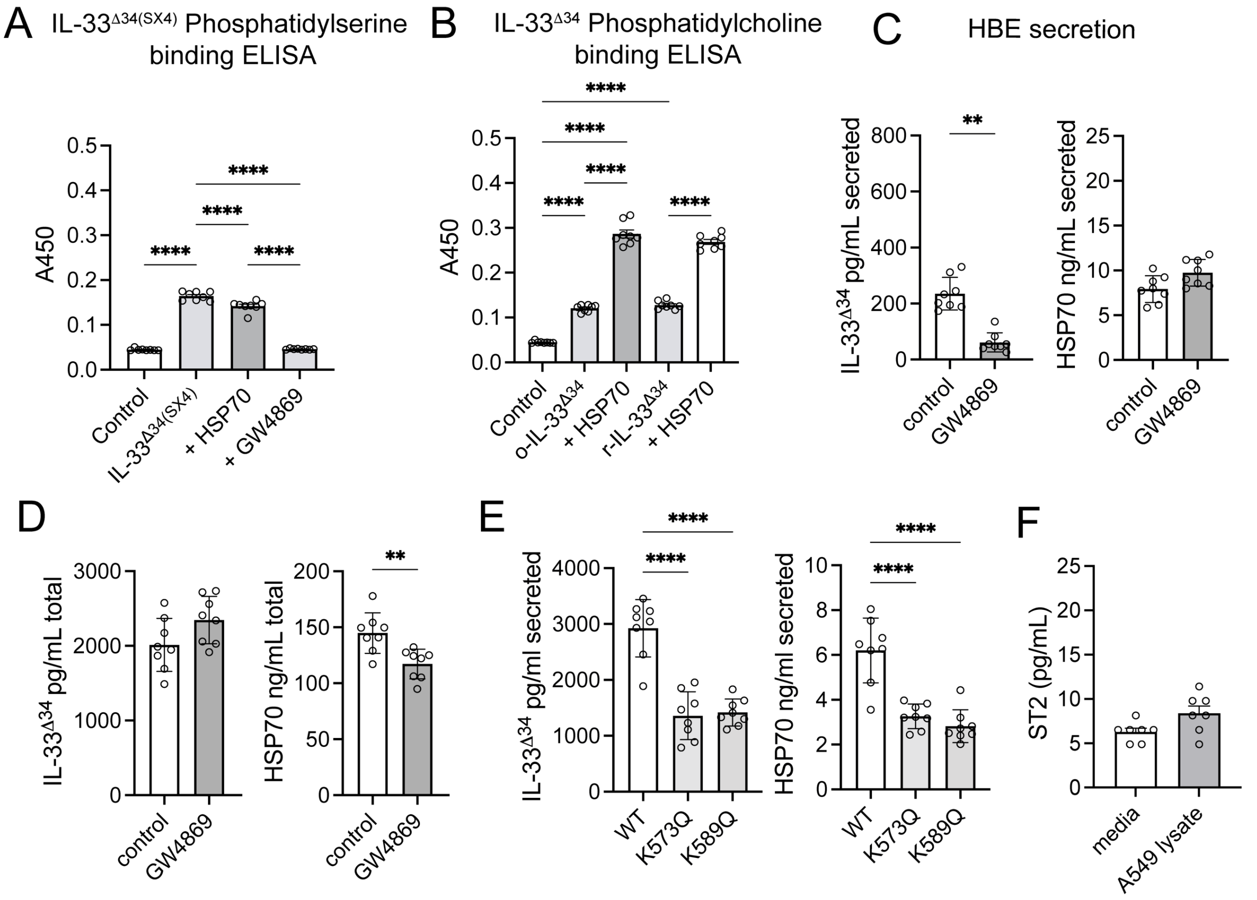

FIGURE S2. Supporting data for Figures 3 and 4. A) Phosphatidylserine (PS) binding for IL-33$^{\Delta34(SX4)}$ mutant (C208S, C227S, C232S, C259S) binding ELISAs under conditions of HSP70 (500 ng/ml) and GW4869 (20 μM) in competition. B) phosphatidylcholine (PC) for oxidized (o-IL-33$^{\Delta34}$) and reduced (r-IL-33$^{\Delta34}$) binding ELISAs under conditions of HSP70 (500 ng/ml) in competition. Detection was performed using biotinylated anti-IL-33 secondary antibody (R&D Systems) and Streptavidin HRP, and TMB substrate followed by A450 absorbance measurements. C, D) Secreted and total Flag-IL-33$^{\Delta34}$-His and endogenous HSP70 in HBE-1 cells under conditions of GW4869 (20 μM) treatment, detection using anti-IL-33 and HSP70 Duosets (R&D Systems) and TMB substrate. E) Secreted Flag-IL-33$^{\Delta34}$-His and HSP70 in conditioned media detected after overexpression of WT or PS-binding deficient mutants K573Q and K589Q. F) ST2 ELISA

performed on A549 lysates to demonstrate minimal receptor protein detected in cellular assay. Assays A-E were conducted in triplicate and with $n = 8$ replicates. Data points displayed with mean ± SEM. Statistical analysis: 1-way ANOVA (A-E); *P*-value limits: $^{*}P < 0.05$, $^{**}P < 0.01$, $^{***}P < 0.001$.

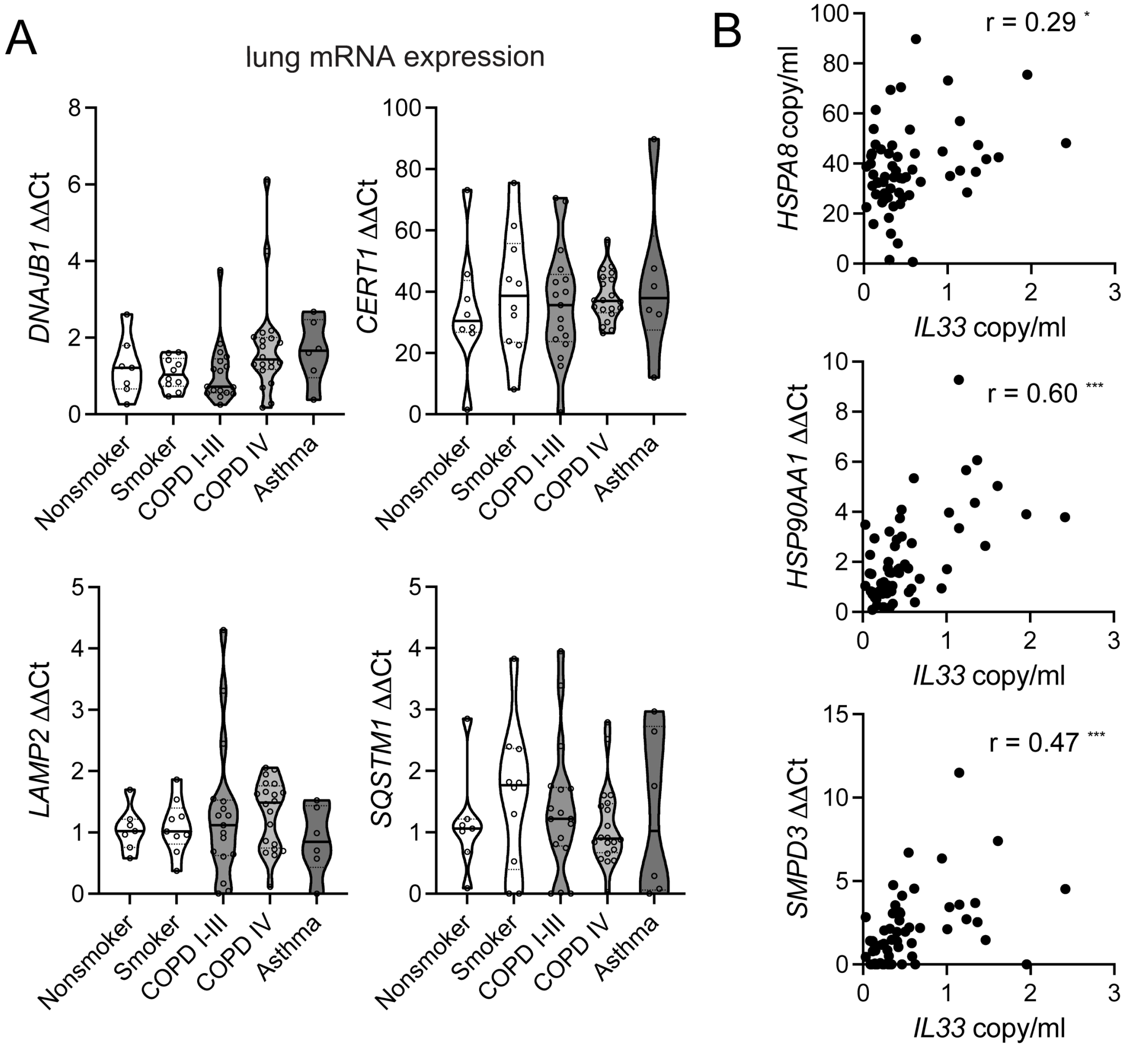

FIGURE S3. Supporting data for Figure 5. A) Lung tissue qPCR for Heat shock and proteostasis intermediates (HSP40/*DNAJB1*, p62/*SQSTM1*) and vesicular trafficking intermediates (*LAMP2*, *CERT1*) in nonsmokers (*n* = 7), smokers (*n* = 11) COPD I-III (*n* = 17), COPD IV (*n* = 20) and severe asthma (*n* = 6) lung tissue specimens represented as fold-change by ΔΔCt method and normalized to GAPDH. B) Pearson's correlation for IL33 expression with HSC70/*HSPA8*, HSP90α/*HSP90AA1* and nSMase2/*SMPD3*.

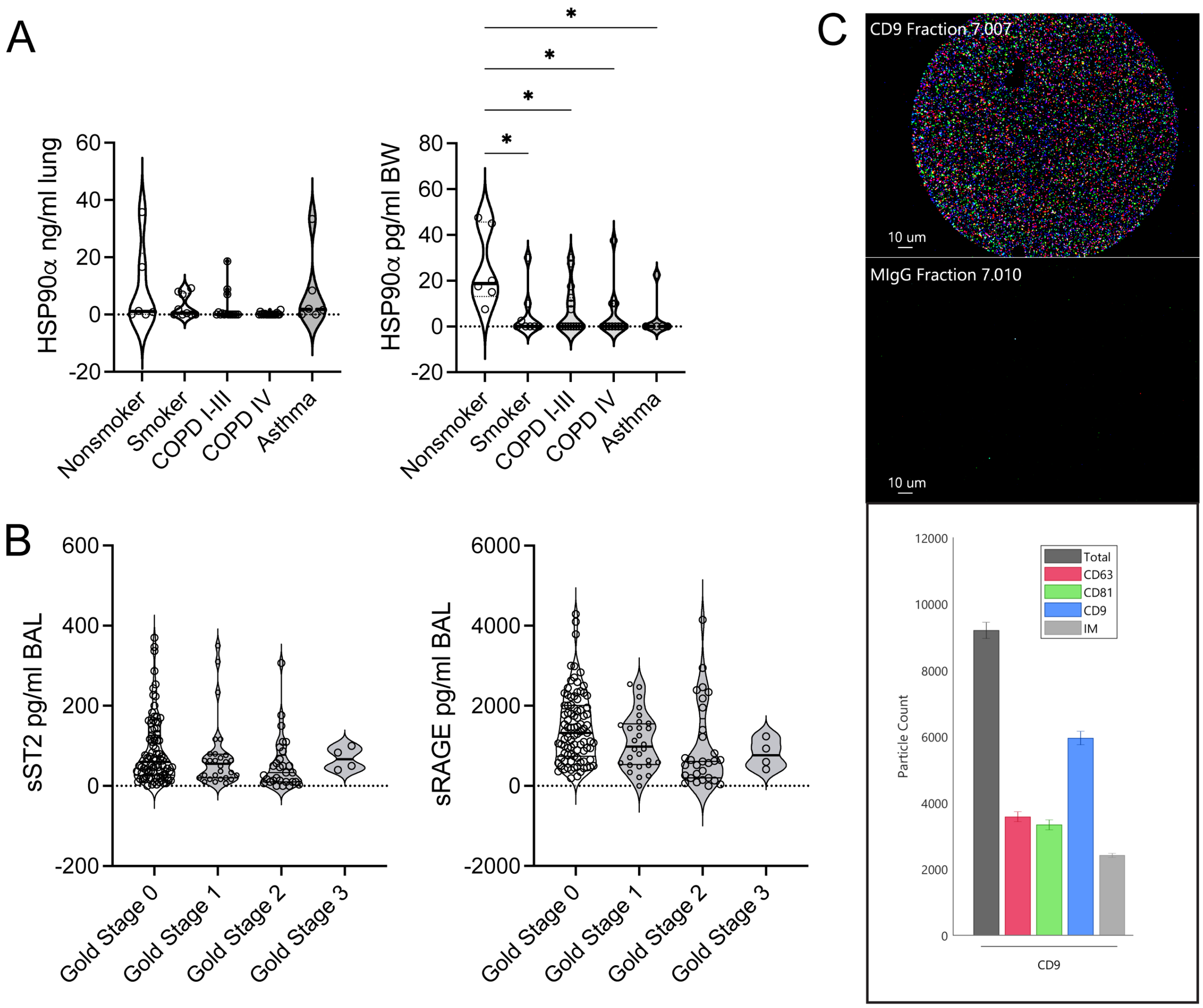

FIGURE S4. Supporting data for Figure 6. A) HSP90α protein levels measured in lung tissue and bronchial wash fluid from representative specimens including nonsmokers (*n* = 7), smokers (*n* = 10) COPD I-III (*n* = 17), COPD IV (*n* = 20) and severe asthma (*n* = 6). B) Soluble ST2 and RAGE protein levels measured by ELISA in SPIROMICS BAL specimens for GOLD Stage COPD 0 (*n* = 91), COPD I (*n* = 28), COPD II (*n* = 29), COPD III (*n* = 4) sample groups. C) Tetraspanin (CD63, CD81 and CD9) positive vesicles were verified using Exoview R100 analysis platform following manufacturer protocols. A representative image of CD9 and negative control capture spots (MIgG, lower subpanel) are represented. Particle counts are averaged from 3 different CD9 capture spots. Data

points displayed as mean ± SEM. Statistical analysis: 1-way ANOVA (A, C); *P*-value limits: *$P < 0.05$.

Table S1. Demographic and clinical data for patient specimens in Washington University cohort: Lung transplant donor specimens without COPD (nonsmoker), rejected donors without COPD (smoker) or mild-moderate (GOLD Stage I-III) COPD[#] and lung transplant recipients with severe (GOLD Stage IV) COPD. [#]Rejected donor lungs grouped as COPD I-III separately from smokers based on the following criteria: 1) presence of emphysema on chest CT, 2) hyperinflation/air trapping on gross pathological examination and 3) positive tobacco history >10 pk-yr. *Severe asthma clinical history with cause of death status asthmaticus.

Characteristics	Nonsmoker	Smoker	COPD I-III[#]	COPD IV	Asthma[*]
Number per group	7	11	17	20	8
Mean age (range)	33 (12-78)	41 (21-67)	56 (21-71)	61 (49-75)	35 (24-44)
Male:Female:Unknown	4:0:3	3:2:6	3:1:10	7:13	2:2:2
FVC (L)	Unknown	Unknown	Unknown	1.9655	Unknown
FEV1 (L)	Unknown	Unknown	Unknown	0.5235	Unknown
FEV_1/FVC (%)	Unknown	Unknown	Unknown	24.95	Unknown
Pack-years (range)	N/A	Unknown	40.8 (30-60)	42.7 (10-96)	Unknown

Table S2. Clinical characteristics of COPD patients based on GOLD Stage obtained from SPIROMICS cohort (3).

Characteristics	COPD 0	COPD I	COPD II	COPD III
Number per group	91	28	29	4
Mean age (range)	56 (40-74)	64 (46-75)	63 (52-74)	54 (41-68)
Male:Female	40:51	16:12	18:11	3:1
FVC (L)	ND	ND	ND	ND
FEV1 (L)	4	2.27	1.53	1.84
FEV_1/FVC (%)	ND	ND	ND	ND
Pack-years (range)	30.9 (0-112)	51.9 (20-118)	58.4 (25.5-108)	58.9 (25-90)

Table S3: List of reagents and materials associated with Methods.

Reagent	Supplier	Reference/ Identifier
**Antibodies and Proteins**		
Human IL-33 rabbit polyclonal (1:50 IF)	Sigma Aldrich	HPA024426
Human IL-33 goat monoclonal (1:1000 WB)	R&D systems	MAB36253
Human IL-33 mouse monoclonal (Nessy-1) (1:1000 IF)	Enzo	ALX-804-840
Human HSP70 antibody (6B3) (1:5000 WB, 1:100 IF)	Cell Signaling	4873S
Human HSP90 antibody (1:100 IF)	Proteintech	60318-1-1g
Soluble human RAGE-Fc	R&D systems	1145-RG
Soluble human ST2	R&D systems	11272-ST
Anti-human Fc	BD	32935S
Anti-rat-HRP	Santa Cruz	sc-2032
Anti-rabbit vectafluor 594 kit	Vector labs	DK-1594
Anti-rat-488	Invitrogen	A21208
IF = immunofluorescence, WB = western blot		
**Commercial lentiviruses**		
Scrambled shRNA	Santa Cruz	sc-108080-V
HSC70 shRNA	Santa Cruz	sc-62655-V
HSP70 shRNA	Santa Cruz	sc-29352-V
**Biological Samples**		
SPIROMICS BAL	NHLBI	N/A
Control and COPD lung tissue	BJH, UNMC	N/A
Control and COPD Bronchial wash fluid	BJH	N/A
**Chemicals, Peptides, and Recombinant Proteins**		
GW4869	Sigma Millipore	D1692
Phosphatidylserine	Avanti Polar lipids	840032P
Phosphatidylcholine	Sigma Aldrich	63556
Pifithrin	Sigma Aldrich	P0122
Gefitinib	Sigma Aldrich	SML1657
Bafilomycin A	Sigma Millipore	B1793-10UG
Rapamycin	Sigma Aldrich	R8781
Geranylgeranylacetone	Sigma Aldrich	G5048
Ivermectin	MedChemExpress	MK-933
Importazole	Calbiochem	401105
**Commercial Assays**		
IL-33 ELISA Duoset	R&D Systems	DY3625
HSP70 ELISA Duoset	R&D Systems	DY1663

HSP90 ELISA	Proteintech	KE00054
ST2	R&D Systems	DY523B
RAGE	R&D Systems	DY1145
**Cell Lines**		
HBE-1	Marisco Lung Institute	HBE1 C1p29
HEK-Blue IL-33 Cells	Invivogen	hkb-hil33
A549 Dual Cells	Invivogen	a549d-nfis
U937	ATCC	CRL-1593.2
**qPCR assays**	IDT PrimeTime® predesigned qPCR assays	
HSPA1A	421177696	
HSPA8	421177700	
IL33	441084120	
STIP1	454622465	
STUB1	454622457	
HSP90AA1	416230057	
SMPD3	218154457	
MUC5AC	166039047	
DNAJB1	424729991	
CERT1	414584019	
LAMP2	206830890	
SQSTM1	454622461	
CCT5	508892978	
CCT7	508892982	
**Constructs**		
human Flag-IL-33$^{\Delta 34}$-His<br>C208S<br>C228S<br>C232S<br>C259S<br>C208/259S<br>C228/232S<br>C208/228/232/259S	pCDH – puro, neo	
human IL-33$^{\Delta 34}$-birA-6His	pET21	
human IL-33$^{\Delta 34}$-MiniTurbo-V5	pCDH - neo	
Human HSP70-His	pCDH - neo	
Human HSP70 (K573Q)-His	pCDH - neo	
Human HSP70 (K589Q)-His	pCDH - neo	

SUPPLEMENTARY REFERENCES

1. Katz-Kiriakos E, Steinberg DF, Kluender CE, Osorio OA, Newsom-Stewart C, Baronia A, et al. Epithelial IL-33 appropriates exosome trafficking for secretion in chronic airway disease. *JCI Insight.* 2021;6(4).
2. Greven JA, and Brett TJ. Production of Eukaryotic Glycoproteins for Structural and Functional Studies Using Expi293F Cells. *Curr Protoc.* 2022;2(8):e512.
3. Couper D, LaVange LM, Han M, Barr RG, Bleecker E, Hoffman EA, et al. Design of the Subpopulations and Intermediate Outcomes in COPD Study (SPIROMICS). *Thorax.* 2014;69(5):492.
