# Supplementary figures and images for: HSP70 is a chaperone for IL-33 activity in chronic airway disease
Source: JCI Insight. 2025 Jun 24;10(15):e193640. doi: 10.1172/jci.insight.193640 (PMC12333954; doi:10.1172/jci.insight.193640)

Figure 1

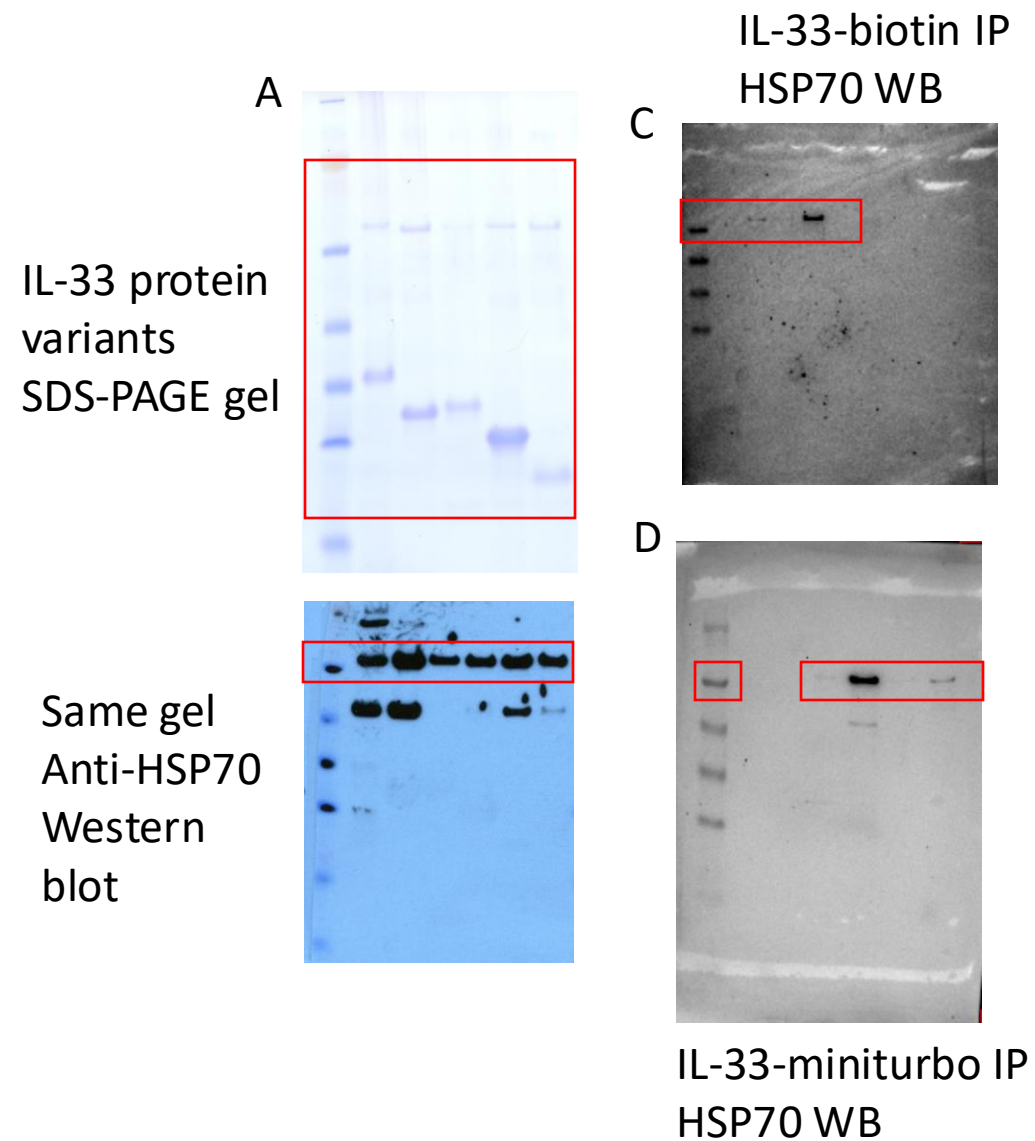

Figure 4

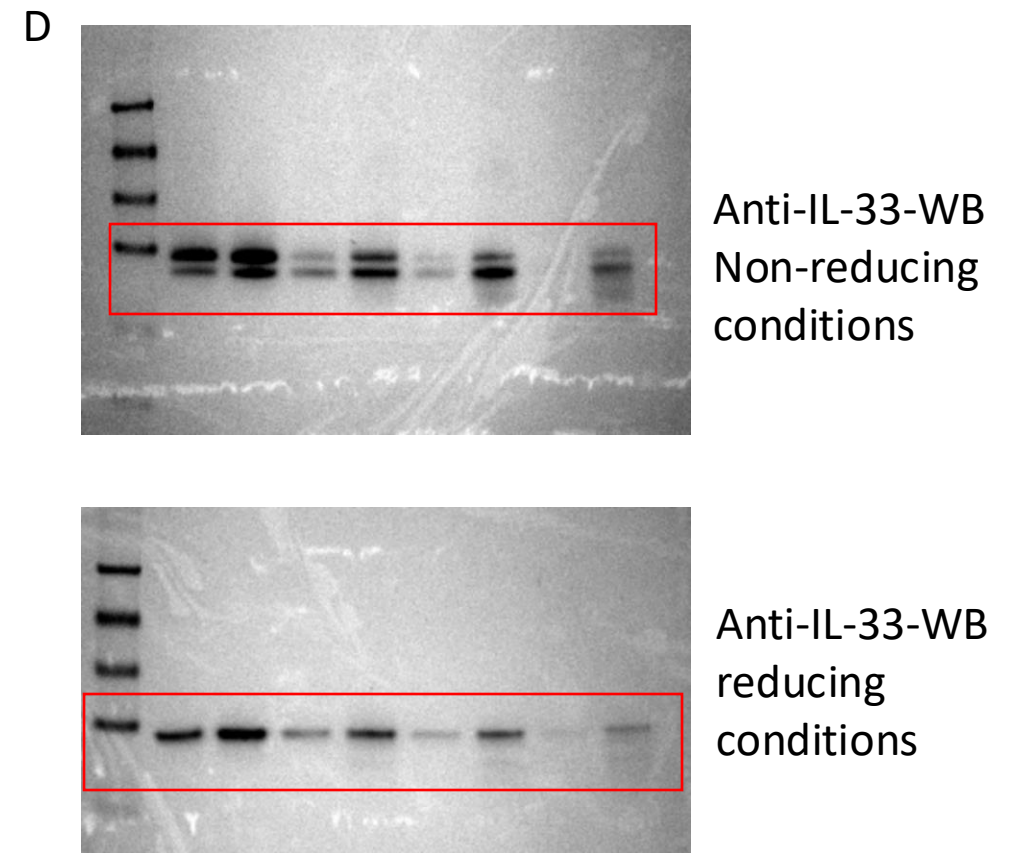

Supplement: Unedited blot and gel images [file jciinsight-10-193640-s066.pdf]
